# A Comment on: Migrating silver eels return from the sea to the river of origin after a false start (2021) by Tambets M *et al*.

**DOI:** 10.1098/rsbl.2022.0202

**Published:** 2022-11-23

**Authors:** Mehis Rohtla, Lauri Saks, Martin Kesler

**Affiliations:** Estonian Marine Institute, University of Tartu, Vanemuise 46a, Tartu, 51003, Estonia

A recently published study [1] investigated the downstream migration of stocked origin silver-stage European eels (*Anguilla anguilla*) and demonstrated that some individuals return from the sea to the river of origin. The authors argue that eels make a ‘false start’, which they interpret as ‘sophisticated strategy’ and ‘important addition to the description of the plastic behaviour of eel’ [1, p. 6]. Eels are indeed very plastic in their behaviour [2], and therefore, the return migration to rivers is plausible. This might be especially so because similar movements to freshwater have been observed in silver-stage American eels (*Anguilla rostrata*), which were otherwise estuary residents according to their otolith chemistry profiles [3,4]. However, here were argue that the interpretations and conclusions of Tambets *et al*. [1] might be premature in the case of European eel, because of an artefact stemming from a peculiar study design. We also provide arguments to support an alternative interpretation for the observed results.

In traditional studies on movement and migration of free-living animals, the study specimens are captured in their natural habitat, tagged, released at the site of capture and then observed to obtain results on movement or migration patterns. Such has been the common practice in silver eel studies (e.g. [5,6]), and such has been the practice of Tambets *et al*. [1] also in their previous studies on other species [7,8]. However, Tambets *et al*. [1] did not follow such practice, because they translocated their study specimens downriver *ca* 250 km from the capture location and released them *ca* 200 m from a hydropower plant intake canal, to study silver eel migration to the sea. We argue that, in the context of the presented study, Tambets *et al*. [1] did not have any biological or other reason to do so, nor did they explain why were the study specimens manipulated to such extent just before the start of their study. In the eel literature, there are also exceptions where study specimens are translocated before the commencement of the study, but these studies have had their specific reasons [9,10]. In an earlier related study [11], Tambets *et al*. estimated the mortality rate of the translocated eels in the Ivangorod hydropower plant turbines. Sublethal effects on the behaviour of eels that have survived the transit are also likely [12], especially because 14% mortality rate was observed in the turbines [11]. Therefore, we propose that the eel return migration to the river up to a year after descent to the sea may also be caused by sublethal injuries that delay the migration and lead to a later return migration to the river.

Tambets *et al*. [1] also claim that they have studied silver eel migration as all eel were silvery in appearance at the time of tagging. They do so without reservation and further discussion on eel developmental stage classification, which is a complex issue [13]. The matter of developmental stage gets even more complicated as stocked individuals were used (that could not imprint their migratory route [14]), navigation out from complex and long freshwater systems could take years [15] and silver eel can revert back to yellow eel [16]. As Tambets *et al*. [1] did not determine eel developmental stage at the time when they returned to the river up to a year after descending to the sea, it cannot be claimed that the eels returning to the river were indeed silver eels.

Tambets *et al*. [1] used Durif′s index [13] to determine eel developmental stage. In their electronic supplementary material, they report that out of the 38 eels, 58% were stage III (i.e. pre-migrant phase), and 42% were stages IV or V (i.e. migrating phases) [1]. Because all eels were sampled in the lake they were stocked to, and not in the river the eels need to migrate into to start their spawning migration, their motivation and/or physiological status for migration most likely differed, and this might also explain why some eels returned to the river after initial descent to the sea. Ambiguity of the developmental stage classifications and/or motivational differences (perhaps owing to translocation?) is clearly visible from data presented by Tambets *et al*. [1] in their table 1 and electronic supplementary material. While 82% of the eels reached Narva Bay during the same autumn as they were captured, 18% reached Narva Bay during the following spring and autumn. However, it should be reminded that these eels did not have to swim *ca* 250 km and the release location was *ca* 200 m from the turbine intake canal, thereby it is likely that many eels just descended with the flow, while being disoriented in the new environment. Furthermore, only eels that reached the sea were included to the study presented by Tambets *et al*. [1]. The original study about eel mortality in turbines contained more eels, some of them died in the turbines and some did not descend at all [11].

In conclusion, the entire interpretive framework of Tambets *et al*. [1] rests on the assumption that the observed behaviour of the stocked and then translocated eels, that also displayed variable developmental stages and had to negotiate hydropower plant turbines, can be used as a proxy for the behaviour of natural silver eel migrants. While the results of Tambets *et al*. [1] study are interesting, at this point, they are only so in a highly experimental and very specific context. We argue that the most parsimonious conclusion for the data presented by Tambets *et al*. [1] is that stocking of juvenile eels to complex and long freshwater systems may cause unorthodox (and possibly maladaptive—as migration is delayed) migration behaviour in eels. This is perhaps even more likely if eels are not allowed to migrate naturally and are translocated before the start of the study. In some of the individuals, the observed return movement to the river might therefore represent a behaviour which is already well known throughout the range—yellow eel inter-habitat shifting between saline and freshwater habitats [2]. This is especially likely, because silver eels can revert back to yellow eel stage during the first part of the migratory phase and continue feeding [16]. We acknowledge that Tambets *et al*. [1] also briefly mention these possibilities in the context of yellow eels, but nevertheless assume that silver eels returned to the river in their study.

However, if silver eel do return to the river origin, instead of the ‘false start’ hypothesis, this behaviour could more precisely represent ‘farewell visits’ (a term coined by Jessop *et al*. [4], which is, admittedly, also not an ideal term describing this phenomenon), representing the need to reorientate their internal magnetic compass. So, a stocked eel deprived from a representative magnetic map, which is naturally obtained by larval imprinting [14], may return to river of origin for an attempt to reorientate itself, perhaps similar to natural migrants if imprinting also occurred in freshwater [4]. However, all this needs to be confirmed with studies on natural and stocked migrants that are allowed to migrate naturally.

## Data Availability

This article has no additional data.
